# Personality and Dietary Intake – Findings in the Helsinki Birth Cohort Study

**DOI:** 10.1371/journal.pone.0068284

**Published:** 2013-07-18

**Authors:** Anna-Maija K. Tiainen, Satu Männistö, Marius Lahti, Paul A. Blomstedt, Jari Lahti, Mia-Maria Perälä, Katri Räikkönen, Eero Kajantie, Johan G. Eriksson

**Affiliations:** 1 Department of Chronic Disease Prevention, National Institute for Health and Welfare, Helsinki, Finland; 2 Unit of General Practice, Helsinki University Central Hospital, Helsinki, Finland; 3 Department of Psychology, Institute of Behavioral Sciences, University of Helsinki, Helsinki, Finland; 4 Children’s Hospital, Helsinki University Central Hospital and University of Helsinki, Helsinki, Finland; 5 Department of General Practice and Primary Health Care, University of Helsinki, Helsinki, Finland; 6 Vasa Central Hospital, Vasa, Finland; 7 Folkhälsan Research Centre, Helsinki, Finland; University of Minnesota, United States of America

## Abstract

**Background:**

Personality traits are associated with health outcomes including non-communicable diseases. This could be partly explained by lifestyle related factors including diet. The personality traits neuroticism, extraversion, openness, agreeableness, and conscientiousness are linked with resilience, meaning adaptability in challenging situations. Resilient people usually comply with favorable health behaviors.

**Objective:**

Our objective was to explore the associations between food and nutrient intake, personality traits and resilience.

**Design:**

A validated semi-quantitative food frequency questionnaire was used to measure diet and the NEO-personality inventory to assess personality in 1681 subjects. Linear regression analysis was used to explore diet-personality associations and cluster analysis to define resilient and non-resilient personality profiles.

**Results:**

Adjusting for age, education and energy intake, and applying Bonferroni corrections, openness in men was associated with higher vegetable (14.9 g/d for 1 SD increase in the personality score, *P_Bonf_* <0.01) and lower confectionery and chocolate (−2.8 g/d, *P_Bonf_* <0.01) intakes. In women, neuroticism was associated with lower fish (−4.9 g/d, *P_Bonf_* <0.001) and vegetable (−18.9 g/d, *P_Bonf_ <*0.01) and higher soft drink (19.9 g/d, *P_Bonf_ <*0.001) intakes. Extraversion, in women, associated with higher meat (5.9 g/d, *P_Bonf_* <0.05) and vegetable (24.8 g/d, *P_Bonf_*<0.001) intakes, openness with higher vegetable (23.4 g/d, *P_Bonf_* <0.001) and fruit (29.5 g/d, *P_Bonf_* <0.01) intakes. Agreeableness was associated with a lower soft drink (−16.2 g/d, *P_Bonf_ <*0.01) and conscientiousness with a higher fruit (32.9 g/d, *P_Bonf_<*0.01) intake in women. Comparing resilient and non-resilient subjects, we found resilience in women to be associated with higher intakes of vegetables (52.0 g/d, *P<*0.001), fruits (58.3 g/d, *P<*0.01), fish (8.6 g/d, *P*<0.01) and dietary fiber (1.6 g/d, *P*<0.01).

**Conclusion:**

Personality traits are associated with dietary intake and especially subjects with resilient personality profiles had healthier dietary intakes. These associations were stronger in women than in men.

## Introduction

Personality traits make up the unique combination of emotional, cognitive, and behavioral traits that characterize an individual and are relatively stable in adulthood [1]. Personality is associated with several health outcomes, which could be explained by behavioral factors associated with personality [2], [3].

The Five-Factor Model (FFM) distinguishes five major personality dimensions [1], [4]: neuroticism, extraversion, openness to experience, agreeableness, and conscientiousness [5], [6], also known as the Big Five. It is among the most commonly used dimensional conceptualizations of personality in research and defines normative personality. The FFM personality traits are associated with BMI [7], [8], diabetes [9], cardiovascular disease [10], and longevity [11], as well as with mortality [12]. Especially conscientiousness (tendency to be dutiful, goal-oriented, persistent) and neuroticism (characterized by anxiety, depression, hostility, impulsivity) have been found to be predictive of health behaviors [2], [3], [13].

To date, only a few studies have explored the associations between personality traits and diet. High neuroticism has been associated with high intakes of sugar and fats [3] and low neuroticism with a Mediterranean style dietary pattern (characterized by vegetables, fish, pasta, oil and vinegar dressing, tomato-based sauces) [7]. High openness (tendency to be open to novelty: ideas, aesthetics, emotions) has also been associated with a Mediterranean style dietary pattern [7], [14], [15]. High agreeableness (tendency to be trustworthy, compliant, and straightforward) and conscientiousness have been associated with high fruit intake [13], and a health aware dietary pattern [7]. High agreeableness is also associated with high vegetable intake [16].

However, personality traits cluster together to form profiles. One of the consistently replicated personality profiles is resilience. Individuals encompassing this profile are low in neuroticism and high in extraversion, openness, agreeableness, and conscientiousness [17], [18], [19]. Generally the concept of resilience encompasses the dynamic processes of adaptation during adverse life events [20], and it has been associated with favorable health behaviors [21] and high intakes of fruits and vegetables [21], [22]. No previous study has tested the associations of resilience and the whole diet.

Our aim was to study the associations between personality traits, resilience, and food and nutrient intake. We hypothesized that healthy dietary habits, such as a high intake of fruits and vegetables, would be associated with low neuroticism and high extraversion, openness, agreeableness and conscientiousness, as well as with resilience.

## Methods

### Ethics Statement

The study was approved by the Ethics Committee of the Hospital District of Helsinki and Uusimaa. All subjects provided written and informed consent.

### Study population

All subjects of the present study were born between 1934 and 1944 in the Helsinki University Central Hospital and had attended child welfare clinics in the city. The participants were part of the Helsinki Birth Cohort Study, originally including 4630 men and 4130 women. In the year 2000, participants who were still alive and residing in Finland were sent questionnaires resulting in replies from 4515 subjects. To obtain a sample size of 2000 or more, 2902 subjects were invited to a clinical study using random-number tables. Of the invited persons, 2003 (928 men and 1075 women) attended a clinical examination between August 2001 and March 2004. The subjects arrived at the study clinic after an overnight fast. Height was measured to the nearest 0.1 cm and weight (in kg) to the nearest 0.1 kg. BMI was calculated by dividing the weight (in kg) by the square of height (in m). Waist circumference was measured midway between the iliac crest and the lowest rib and recorded to the nearest 0.1 cm. Hip circumference was measured at the maximum level of the trochanters and recorded similarly. Education level was used as an indicator of socioeconomic status and the division of subjects to the educational groups used was as follows: <12y *n* = 542 and ≥12y *n* = 1133.

### Dietary assessment

The whole diet was assessed by using a validated, self-administered semi-quantitative 128-item food frequency questionnaire (FFQ) containing 12 different food groups e.g. vegetables, and milk products [23], [24]. The FFQ was designed to measure the ordinary diet over the previous 12 months. There were 9 frequency categories ranging from never or seldom to ≥6 times/day. The subjects completed the FFQ at the clinic and it was checked by a study nurse. The food intake data were entered and processed at the National Institute for Health and Welfare using the National Food Consumption Database FINELI® (National Institute for Health and Welfare, Helsinki, Finland) [25]. Subjects were excluded if their calculated energy intake was <650 or >6100 kcal/d, which corresponded to 0.5% at each end of the self-reported energy intake scale (*n* = 22).

### Personality assessment

We used the Finnish version [26] of the NEO-PI (Neuroticism-Extroversion-Openness Personality Inventory) [5] questionnaire to measure the FFM personality dimensions. The NEO-PI is a 181-item personality inventory [5], [26] consisting of 48 items for neuroticism, extraversion, and openness to experience and 18 items for agreeableness and conscientiousness. The five response options on a Likert scale vary between untrue and extremely true. Thus, a continuous score is yielded for each subject. Personality scales were standardized by sex and differences in personality score were expressed for 1 SD. Usually, the distributions of the personality scales are normally distributed, but this applied only for extraversion, openness to experience, and agreeableness in this study. The distributions of conscientiousness and neuroticism were moderately negatively and moderately positively skewed, and therefore, we transformed these scales with squared and square root transformations, respectively, to reach normal distribution. The NEO-PI questionnaire was mailed between April 2004 and June 2005, on average two years after the clinical examination when dietary intake was assessed, to those participants who were still traceable (*n* = 1975) [27]. In all, 1701 subjects completed the questionnaire. Five subjects were excluded because they indicated not having answered the inventory truthfully and another 15 subjects were excluded, because they had missing data on socioeconomic data. In all, 1681 subjects (938 women and 743 men) had valid data available on both personality and diet. One subject only had data on agreeableness; therefore, the cluster analysis only includes data of 1680 subjects.

### Statistical analysis

We performed multiple linear regression analysis to explore the associations between foods and nutrients (as dependent variables) and personality traits (as independent variables). We analyzed the whole sample testing for gender interactions, and found several interactions regarding e.g. neuroticism and fats, extraversion and vegetables, and conscientiousness and soft drinks. Thus, we decided to analyze men and women separately. This was further justified by the fact that dietary intakes usually differ by gender. Model 1 was adjusted for age, educational attainment (<12 years vs. ≥12 years of education), and total energy intake. In model 2, we added BMI and WHR. We calculated the individual results in foods and nutrient intakes as continuous variables (g/d, mg/d, or μg/d) for 1 SD increase of each personality trait. When analyzing the nutrients we adjusted for total energy intake by using residuals [28]. To address the problem of multiple testing, we applied Bonferroni corrections. After this correction the new threshold for statistical significance was set at 0.01 (0.05/5 personality traits) concerning the analyses of the five separate personality traits. To define resilience, we applied Wards hierarchial clustering procedure. We applied linear regression analysis to explore the associations of the clusters (as independent variables) and food and nutrient intake (as dependent variables), adjusting for the same covariates as in models 1 and 2. Statistical tests were considered significant if *P*<0.05. The analyses were performed using R software (version 2.13.1) R Foundation for Statistical Computing, Vienna, Austria and Stata® software (release 11), Stata Corporation, College Station, Texas, USA.

## Results

### Personality traits and dietary intake

**Table 1** shows the characteristics of the study population. Mean age was 61.5 y in both men (*n* = 743) and women (*n* = 938). After adjusting for age, educational attainment and total energy intake and applying Bonferroni corrections, openness was associated with higher vegetable (14.9 g/d for every 1 SD increase in the personality score, *P_Bonf_* <0.05, **Table 2**) and lower confectionery and chocolate (−2.8 g/d, *P_Bonf_* <0.05) intakes in men.

**Table 1:** Characteristics of the study population.

	Men (*n* = 743)		Women (*n* = 938)	
	Mean	SD	Mean	SD
Age (y)	61.5	2.8	61.5	3.1
Secondary education or higher (%)	73.6		62.5	
BMI (kg/m^2^)	27.5	4.3	27.7	5.0
Neuroticism	65.4	26.6	74.1	27.3
Extraversion	103.8	23.3	102.8	23.9
Openness	103.4	21.3	113.8	21.7
Agreeableness	47.2	7.7	51.1	7.6
Conscientiousness	50.6	9.6	50.6	9.8
Resilience^1^ (%)	49.9		48.4	
Energy (kcal/d)	2482	877	2043	693
Protein (g/d)	87.2	13.1	89.7	12.9
Fat (g/d)	92.7	39.3	77.1	12.5
Carbohydrates (g/d)	236.6	34.5	248.4	34.7
Alcohol (g/d)	13.3	14.9	5.7	8.0

1 By resilience is meant the personality profile with low neuroticism and high extraversion, openness to experience, agreeableness and conscientiousness.

**Table 2:** Linear regressions between foods as dependent variables and the personality traits neuroticism, extraversion, openness to experience, agreeableness and conscientiousness as independent variables in men (*n* = 743).

		Neuroticism		Extraversion		Openness		Agreeableness		Conscientiousness	
Foods (g/d)		Beta^1^	*P*-value^2^	Beta^1^	*P*-value^2^	Beta^1^	*P*-value^2^	Beta^1^	*P*-value^2^	Beta^1^	*P*-value^2^
Cereals		−1.9	0.41	−1.2	0.61	1.8	0.42	4.2	0.06	0.5	0.83
Milk and dairy products		6.3	0.61	−21.4	0.08	−6.9	0.59	23.8	0.05^3^	−15.9	0.20
	Milks	13.1	0.22	−11.2	0.29	−18.3	0.10	6.3	0.54	−17.6	0.10
	Sour milk products	−7.9	0.23	−9.6	0.15	9.6	0.17	17.4	<0.01^3,4^	3.5	0.60
Fats		−1.0	0.09	0.6	0.36	−0.4	0.53	−0.7	0.27	1.0	0.10
	Butter	−0.4	0.20	0.1	0.79	−0.4	0.22	0.05	0.84	0.0	0.86
	Margarine	0.0	0.93	−0.2	0.63	0.2	0.71	0.3	0.45	0.5	0.17
Meat and meat products		0.1	0.98	5.4	0.15	−4.3	0.27	−4.9	0.18	1.3	0.72
	Red meat	0.8	0.81	3.3	0.35	−6.1	0.10	−5.7	0.10	1.5	0.67
	Processed meat	1.0	0.68	−4.4	0.06	−3.3	0.18	−1.4	0.55	0.64	0.78
Fish		−0.7	0.69	2.1	0.26	2.4	0.21	0.8	0.67	0.1	0.95
Vegetables^5^		0.2	0.97	11.8	<0.05	14.9	<0.01^3,4^	−6.5	0.22	4.5	0.41
Fruits^6^		−8.6	0.44	15.1	0.18	26.7	<0.05^3^	4.8	0.66	7.1	0.52
Sugar and confectionery		−1.0	0.34	−0.4	0.73	−2.1	0.06	−1.3	0.23	2.1	0.05
	Confectionery and chocolate	−0.5	0.59	−0.4	0.71	−2.8	<0.01^3,4^	−1.7	0.09	2.0	<0.05
Soft drinks		−3.7	0.64	0.4	0.96	−20.2	<0.05^3^	−15.2	0.06	16.4	<0.05

1 Beta coefficient for the change in food intake (g/d) for every 1 SD increase in the personality score.

2 Model 1 adjusted for age, educational attainment, and total energy intake.

3 Remained significant after applying Model 2 adjusted for age, educational attainment, total energy intake, BMI and WHR.

4 Remained significant after applying the Bonferroni corrections.

5 Potatoes not included.

6 Berries and fruit juice included.

In women, neuroticism was associated with lower vegetable (−18.9 g/d, *P_Bonf_* <0.01, **Table 3** **)** and fish (−4.9 g/d, *P_Bonf_ <*0.001) and higher soft drink (19.9 g/d, *P_Bonf_ <*0.001) intakes. Extraversion was associated with higher meat and meat product (5.9 g/d, *P_Bonf_ <*0.05), and vegetable (24.8 g/d, *P_Bonf_* <0.001) intakes. Regarding openness, women reported a lower processed meat (−3.5 g/d, *P_Bonf_* <0.01) intake and higher vegetable (23.4 g/d, *P_Bonf_* <0.01) and fruit (29.5 g/d, *P_Bonf_* <0.01) intakes. In women, agreeableness was associated with lower intakes of meat and meat products (−6.0 g/d, *P_Bonf_* <.01) and soft drinks (−16.2 g/d, *P_Bonf_ <*0.01) and conscientiousness was associated with a higher intake of fruits (32.9 g/d, *P_Bonf_ <*0.01).

**Table 3:** Linear regressions between foods as dependent variables and the personality traits neuroticism, extraversion, openness to experience, agreeableness and conscientiousness as independent variables in women (*n* = 938).

		Neuroticism		Extraversion		Openness		Agreeableness		Conscientiousness	
Foods (g/d)		Beta^1^	*P*-value^2^	Beta^1^	*P*-value^2^	Beta^1^	*P*-value^2^	Beta^1^	*P*-value^2^	Beta^1^	*P*-value^2^
Cereals		3.2	0.05	−7.6	<0.001^3,4^	−2.0	0.25	3.8	<0.05^3^	−3.7	<0.05^3^
Milk and dairy products		−5.5	0.5	−10.3	0.26	−3.3	0.73	6.9	0.4	8.7	0.3
	Milks	14.7	0.05	−13.8	0.07	−11.0	0.17	−12.2	0.1	−5.9	0.4
	Sour milk products	−18.4	<0.01 3,4	−0.4	0.95	6.0	0.36	17.8	<0.01^3,4^	14.6	<0.05
Fats		0.6	0.2	−0.4	0.39	−0.6	0.25	−0.3	0.5	−0.6	0.2
	Butter	0.01	1.0	−0.05	0.82	−0.1	0.74	−0.08	0.7	−0.4	<0.05^3^
	Margarine	0.7	<0.05^3^	−1.0	<0.01^3,4^	−0.9	<0.01^3,4^	−0.3	0.3	−0.5	0.08
Meat and meat products		−1.3	0.6	5.9	<0.05^4^	−2.6	0.29	−6.0	<0.01^4^	0.2	0.9
	Red meat	0.08	1.0	4.2	<0.05	−4.7	<0.05^3^	−4.9	<0.05^4^	−1.6	0.4
	Processed meat	2.2	<0.05	0.2	0.86	−3.5	<0.01^3,4^	−2.7	<0.01^4^	−2.3	<0.05
Fish		−4.9	<0.001^3,4^	2.0	0.17	1.9	0.22	0.2	0.9	3.5	<0.05^3^
Vegetables^5^		−18.9	<0.01^3,4^	24.8	<0.001^3,4^	23.4	<0.001^3,4^	11.4	0.08	16.2	<0.05^3^
Fruits^6^		−17.5	0.1	11.7	0.27	29.5	<0.01^3,4^	15.5	0.1	32.9	<0.01^3,4^
Sugar and confectionery		1.1	0.1	−0.9	0.15	−1.3	0.06	−0.5	0.5	−1.3	0.05^3^
	Confectionery and chocolate	1.1	0.06	−0.4	0.47	−1.0	0.07	−1.0	0.06	−1.3	<0.05^3^
Soft drinks		19.9	<0.001^3,4^	2.1	0.72	−11.9	0.06	−16.2	<0.01^3,4^	−4.0	0.5

1 Beta coefficient for the change in food intake (g/d) for every 1 SD increase in the personality score.

2 Model 1 adjusted for age, educational attainment, and total energy intake.

3 Remained significant after applying Model 2 adjusted for age, educational attainment, total energy intake, BMI and WHR.

4 Remained significant after applying the Bonferroni corrections.

5 Potatoes not included.

6 Berries and fruit juice included.

Regarding nutrients and after adjusting for age, educational attainment and total energy intake, openness was associated with higher dietary fiber (0.8 g/d, *P_Bonf_* <0.05, **Table 4**) carotene (645.2 μg/d, *P_Bonf_* <0.01) and vitamin C (13.8 mg/d, *P_Bonf_*<0.001) intakes in men. In women, neuroticism was associated with lower intakes of protein (−1.2 g/d, *P_Bonf_* <0.05, **Table 5**), and vitamin C (−13.5 mg/d, *P_Bonf_*<0.01). Extraversion on the other hand was, in women, associated with higher protein (1.3 g/d, *P_Bonf_*<0.05) and lower carbohydrate (−4.0 g/d, *P_Bonf_* <0.01) intakes. In women, openness was associated with higher dietary fiber (1.2 g/d, *P_Bonf_* <0.001), carotene (892.3 μg/d, *P_Bonf_*<0.01) and vitamin C (14.3 mg/d, *P_Bonf_*<0.01) intakes. Women with higher agreeableness reported higher dietary fiber intake (1.4 g/d, *P*<0.001), and lower alcohol intake (−1.1 g/d, *P_Bonf_* <0.001). In women, conscientiousness was associated with higher intakes of sugars (3.9 g/d, *P_Bonf_* <0.001) and vitamin C (14.2 mg/d, *P_Bonf_* <0.001). Neuroticism was in women associated with lower intakes of several B vitamins, but the four other traits were associated with higher intakes of many B vitamins. Adding BMI and WHR to the model did not markedly change the significance levels.

**Table 4:** Linear regressions between nutrients as dependent variables and the personality traits neuroticism, extraversion, openness to experience, agreeableness and conscientiousness as independent variables in men (*n* = 743).

		Neuroticism		Extraversion		Openness		Agreeableness		Conscientiousness	
Nutrients^1^		Beta^2^	*P*-value^3^	Beta^2^	*P*-value^3^	Beta^2^	*P*-value^3^	Beta^2^	*P*-value^3^	Beta^2^	*P*-value^3^
Energy (kcal/d)		43.2	0.19	63.7	0.05	69.0	<0.05^4^	−44.4	0.17	−39.4	0.23
Protein (g/d)		−0.2	0.72	0.8	0.11	0.2	0.69	0.1	0.79	−0.2	0.71
Carbohydrate (g/d)		−1.5	0.26	−0.9	0.46	1.3	0.33	2.6	**<**0.01^4,5^	1.3	0.32
	Sugars (g/d)	−1.5	0.19	0.5	0.67	2.3	0.05	1.3	0.25	0.3	0.77
	Dietary fiber (g/d)	−0.06	0.84	0.2	0.39	0.8	<0.01^4,5^	0.5	0.09	0.3	0.23
Fat (g/d)		0.006	0.99	−0.2	0.72	−1.1	<0.05^4^	−1.0	**<**0.05^4^	0.1	0.83
	SAFA (g/d)	0.01	0.97	−0.3	0.11	−0.6	<0.01^4,5^	−0.3	0.20	−0.1	0.59
	Marine n−3 (g/d)	−0.03	0.27	0.05	<0.05	0.02	0.47	0.005	0.83	0.01	0.63
Alcohol (g/d)		1.0	0.08	0.3	0.55	0.5	0.35	−0.4	0.52	−0.9	0.10
Carotens (µg/d)		48.3	0.79	370.5	<0.05	645.2	<0.001^4,5^	19.9	0.91	79.0	0.65
Vitamin C (mg/d)		−3.4	0.29	8.6	<0.01^4,5^	13.8	<0.001^4,5^	2.0	0.54	0.4	0.90
Thiamine (mg/d)		−0.01	0.46	0.05	<0.001^4,5^	0.00	0.82	0.02	0.29	0.02	0.29
Niacin (mg/d)		−0.1	0.55	0.7	<0.01^4,5^	0.3	0.20	0.09	0.66	−0.06	0.77
Pyridoxine (mg/d)		−0.03	0.16	0.07	<0.01^4,5^	0.03	0.12	0.01	0.57	0.02	0.46
Iron (mg/d)		−0.04	0.64	0.3	<0.01^4,5^	0.3	<0.01^4,5^	0.03	0.75	0.08	0.36
Potassium (mg/d)		18.8	0.53	43.5	0.14	74.2	<0.05^4^	16.5	0.57	−11.3	0.70
Calcium (mg/d)		−0.9	0.95	−26.4	0.05	12.8	0.37	28.1	**<**0.05^4^	−21.0	0.12
Magnesium (mg/d)		1.3	0.61	3.6	0.16	8.5	<0.01^4,5^	4.8	0.06	−1.8	0.49
Sodium (mg/d)		2.3	0.91	25.5	0.20	6.4	0.76	−15.8	0.42	8.2	0.68
Zink (mg/d)		−0.02	0.86	0.1	0.07	0.1	0.19	0.09	0.28	0.01	0.89

1 Nutrients adjusted for energy using the residual method.

2 Beta coefficient for the change in nutrient intake (g/d, mg/d, or μg/d) for every 1 SD increase in the personality score.

3 Model 1 adjusted for age, educational attainment, and total energy intake.

4 Remained significant after applying Model 2 adjusted for age, educational attainment, total energy intake, BMI and WHR.

5 Remained significant after applying the Bonferroni corrections.

Mean difference in food use (g/d) is given for the increase of 1 SD in the personality score.

**Table 5:** Linear regressions between nutrients as dependent variables and the personality traits neuroticism, extraversion, openness to experience, agreeableness and conscientiousness as independent variables in women (*n* = 938).

		Neuroticism		Extraversion		Openness		Agreeableness		Conscientiousness	
Nutrients^1^		Beta^2^	*P*-value^3^	Beta^2^	*P*-value^3^	Beta^2^	*P*-value^3^	Beta^2^	*P*-value^3^	Beta^2^	*P*-value^3^
Energy (kcal/d)		32.4	0.16	46.3	<0.05^4^	48.5	0.05	−11.2	0.63	−49.0	<0.05^4^
Protein (g/d)		−1.2	<0.01^4,5^	1.3	<0.01^4,5^	0.8	0.06	0.3	0.55	−0.04	0.9
Carbohydrate (g/d)		−0.2	0.90	−4.0	<0.001^4,5^	−0.4	0.72	3.8	<0.01^4,5^	2.3	<0.05
	Sugars (g/d)	−2.7	<0.05^4^	0.8	0.47	1.4	0.22	1.6	0.14	3.9	<0.001^4,5^
	Dietary fiber (g/d)	−0.6	0.05	−0.3	0.37	1.2	<0.001^4,5^	1.4	<0.001^4,5^	0.2	0.48
Fat (g/d)		0.4	0.36	0.8	0.06	−0.2	0.60	−1.2	<0.01^4,5^	−0.9	<0.05
	SAFA (g/d)	0.3	0.22	0.3	0.20	−0.3	0.15	−0.5	<0.05^4^	−0.4	<0.05^4^
	Marine n−3 (g/d)	−0.1	<0.01^4,5^	0.1	<0.01^4,5^	−0.04	0.04	−0.008	0.66	0.06	<0.01^4,5^
Alcohol (g/d)		0.4	0.09	0.6	<0.05^4^	−0.1	0.67	−1.1	<0.001^4,5^	−0.4	0.13
Carotens (µg/d)		−704.2	<0.01^4,5^	654.4	<0.01^4,5^	892.3	<0.001^4,5^	615.5	<0.05^4,5^	424.4	0.07
Vitamin C (mg/d)		−13.5	<0.001^4,5^	10.4	<0.01^4,5^	14.3	<0.001^4,5^	6.7	0.07	14.2	<0.001^4,5^
Thiamine (mg/d)		−0.03	<0.01^4,5^	−0.003	0.81	0.03	<0.05^4^	0.05	<0.001^4,5^	0.03	<0.05^4^
Niacin (mg/d)		−0.7	<0.001^4,5^	0.7	<0.01^4,5^	0.7	<0.001^4,5^	0.3	0.18	0.32	0.09
Pyridoxine (mg/d)		−0.1	<0.001^4,5^	0.06	<0.01^4,5^	0.1	<0.001^4,5^	0.05	<0.05^4,5^	0.08	<0.001^4,5^
Iron (mg/d)		−0.2	0.07	0.02	0.83	0.2	<0.05^4^	0.3	<0.001^4,5^	0.04	0.61
Potassium (mg/d)		−112.4	<0.001^4,5^	51.3	0.08	89.0	<0.01^4,5^	72.5	<0.05^4,5^	89.8	<0.01^4,5^
Calcium (mg/d)		−21.2	0.09	7.3	0.57	12.5	0.36	20.8	0.10	13.0	0.30
Magnesium (mg/d)		−4.2	0.07	−0.2	0.92	8.0	<0.01^4,5^	8.7	<0.001^4,5^	2.0	0.38
Sodium (mg/d)		20.3	0.27	24.8	0.18	10.6	0.58	−11.5	0.53	−31.1	0.08
Zink (mg/d)		−0.2	<0.01^4,5^	0.1	0.15	0.2	0.01^4^	0.24	<0.01^4,5^	−0.02	0.78

1 Nutrients adjusted for energy using the residual method.

2 Beta coefficient for the change in nutrient intake (g/d, mg/d, or μg/d) for every 1 SD increase in the personality score.

3 Model 1 adjusted for age, educational attainment, and total energy intake.

4 Remained significant after applying Model 2 adjusted for age, educational attainment, total energy intake, BMI and WHR.

5 Remained significant after applying the Bonferroni corrections.

Mean difference in food use (g/d) is given for the increase of 1 SD in the personality score.

### Resilience and dietary intake

The cluster analysis yielded two groups: the resilient (*n* = 855, 371 men and 454 women) and non-resilient (*n* = 825, 371 men and 484 women) personality profiles. Compared to non-resilient subjects, resilient subjects had lower scores on neuroticism (Mean Difference (MD) in SD units  = −1.36, *P*<0.001), and higher scores on extraversion (MD = 0.47, *P*<0.001), openness (MD = 0.24, *P*<0.001), agreeableness (MD = 1.02, *P*<0.001), and conscientiousness (MD = 1.13, *P*<0.001).

There was no difference in age between the subjects with resilient and non-resilient personality profiles, while BMI was lower in resilient than in non-resilient subjects (Mean values  = 27.4 kg/m^2^ and  = 27.9 kg/m^2^, *P<*0.05). The mean energy intakes for resilient and non-resilient subjects were 2220.9 kcal/d and 2253.5 kcal/d, respectively (*P* = 0.40).

**Figure 1:**
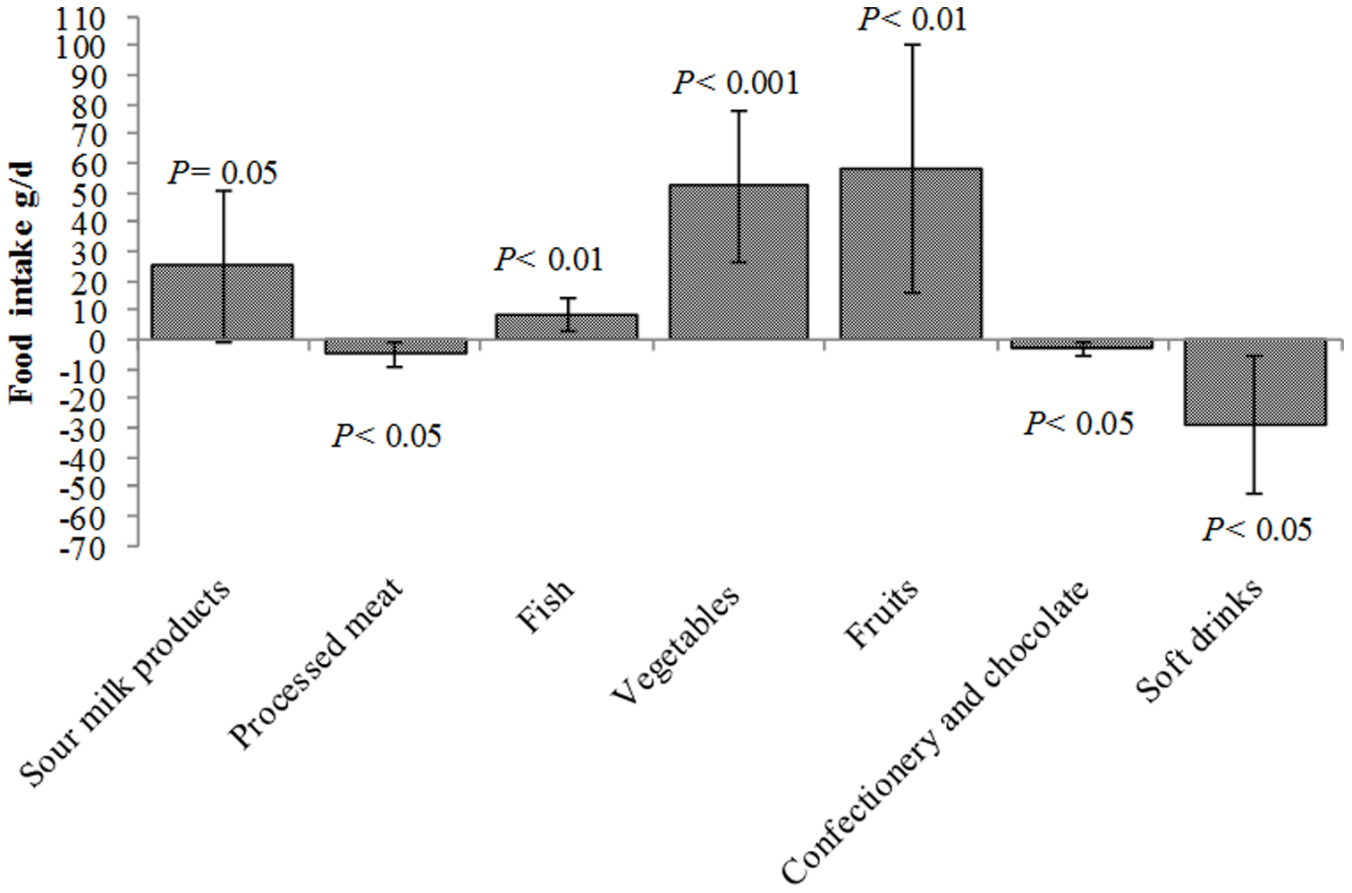
Differences in food intake in resilient women (*n* = 454) compared to non-resilient women (*n* = 484). Values are given as g/d and adjusted for age, educational attainment, and total energy intake. The error bars indicate 95% confidence intervals. Resilience is defined as a personality profile with low neuroticism and high extraversion, openness to experience, agreeableness and conscientiousness. Vegetables do not include potatoes. Fruits include fruits, berries and fruit juices.

**Figure 2:**
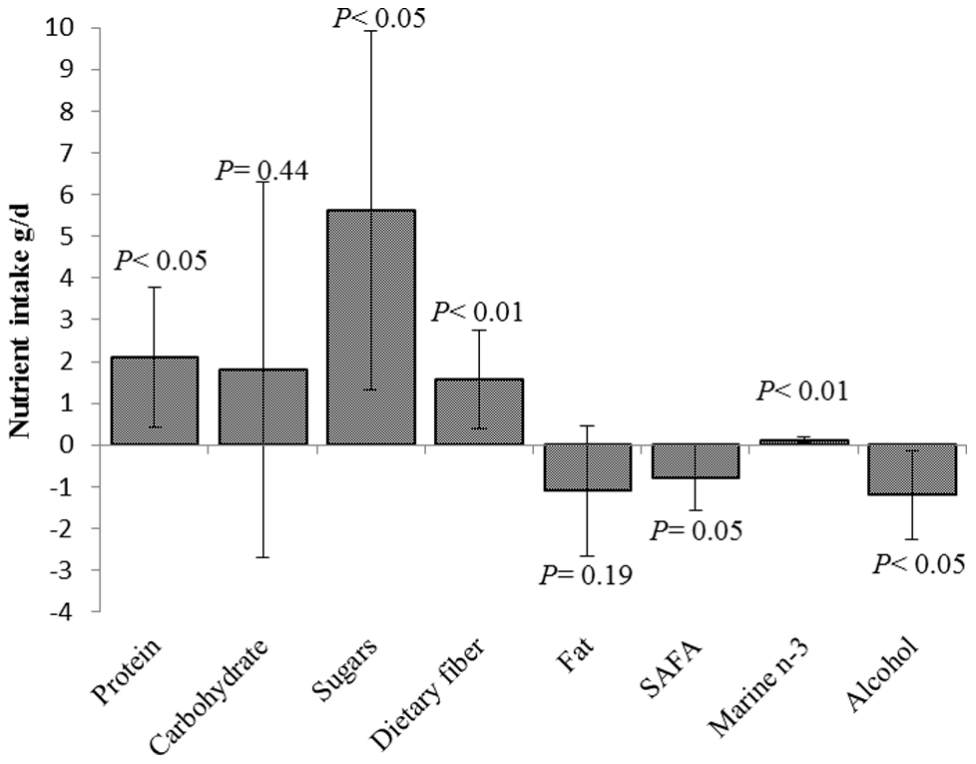
Differences in nutrient intake in resilient women (*n* = 454) compared to non-resilient women (*n* = 484). Values are given as g/d and adjusted for age, educational attainment, and total energy intake. The error bars indicate 95% confidence intervals. Nutrients are adjusted for energy by using the residual method. Resilience is defined as a personality profile with low neuroticism and high extraversion, openness to experience, agreeableness and conscientiousness.

There were no statistically significant results for men concerning resilience and food intake (all *P*-values ≥0.11). Women with resilient personality profiles reported significantly higher intakes of vegetables (52.0 g/d, *P<*0.001, **Figure 1**) and fruits (58.3 g/d, *P<*0.01). Other favorable food choices for resilient women included lower intakes of processed meat products (−4.9 g/d, *P<*0.05) and soft drinks (−29.3 g/d, *P*<0.05) and a higher intake of fish (8.6 g/d, *P<*0.01). Regarding nutrients, resilient men reported higher vitamin C intakes (12.8 mg/d, *P*<0.05), but there were no statistically significant results concerning all the other nutrients (all *P*- values ≥0.10). Resilient women had higher intakes of e.g. protein (2.1 g/d, *P*<0.05, **Figure 2**), dietary fiber (1.6 g/d, *P*<0.01), and sugars (5.6 g/d, *P<*0.05), and lower intakes of alcohol (−1.2 g/d, *P<*0.05). Adding the covariates BMI and WHR to the model did not substantially change the results (data not shown).

## Discussion

We set out to study the associations between personality traits, resilience and food and nutrient intake in 1681 Finns in late adulthood. As hypothesized, neuroticism was associated with mainly poorer dietary quality as the intakes of fish and vegetables were lower and intake of soft drinks was higher, but this applied to women only. In line with our hypothesis, we observed extraversion being e.g. associated with a higher vegetable intake in women. Openness was associated with higher intakes of vegetables and fruits in both genders. Agreeable women showed favorable trends, as did conscientious women, the latter reporting e.g. a higher fruit intake. Some of these trends were further strengthened when testing subjects with resilient vs. non-resilient personality profiles; resilient women reported higher intakes of vegetables, fruits, fish, and dietary fiber and lower intake of alcohol. Our results were in line with our original study hypothesis and the associations were not due to age, educational attainment, or total energy intake.

The fact that statistically significant findings were scarce in men could perhaps be explained by the spouses’ personality having more of an influence for men than for women, as wives in the generation studied, are usually responsible for cooking. However, in a previous study only openness showed an association when comparing each participant’s personality traits with their own as well as their spouses’ dietary consumption measured by the Modified Healthy Eating Index [15]. The results in conscientious men were surprising, as they reported higher intakes of sugar and confectionery and soft drinks. This was contrary to our hypothesis and the results in women.

Our result that neuroticism was associated with several unfavorable dietary intakes is consistent with two previous cross-sectional studies. One study in Japanese students found neuroticism to be associated with intakes of sweet and salty foods [29] while a Scottish study found [7], high neuroticism to be associated with a traditional convenience diet (eating more tinned vegetables, meat pies, pasties and sausage rolls, puddings etc.) and low neuroticism to be associated with a Mediterranean-style diet. A recent Estonian study [14] also found low neuroticism to be associated with a health aware dietary pattern. Our results are also in line with findings showing that high neuroticism is associated with obesity [8], metabolic syndrome [30] and an increased risk for CVD [30], [31].

Our finding that extraversion is in women associated with a higher vegetable intake is in line with previous findings in which high extraversion is associated with higher scores on a health aware dietary pattern [14] and a Mediterranean style diet [7]. However, the finding that extraversion is associated with higher meat and meat product intake, is contradictory to our hypothesis. This finding could be explained by extraverts regularly attending social situations, where meat is often abundant on the menu and the possibilities to choose what to eat are limited.

The present result of openness being associated with higher vegetable and fruit and lower processed meat intakes could be explained by the tendency of open people to readily adopt modern trends, such as healthy eating. In the present study, the magnitude of the combined fruit and vegetable intake in women was substantial as it summed up to +52.9 g/d for every 1 SD increase in openness. Thus, the intakes of subjects at the opposite ends of the normal distribution scale (with a difference of 6 SD), differ as much as 317.4 g/d. In line with our findings on openness are previous studies, which found high openness to be associated with a Mediterranean style diet [7], a health aware dietary pattern [14] as well as the Modified Healthy Eating Index [15] and low openness to be associated with a preference for sweet foods [7]. Thus, openness appears to be the personality trait most consistently associated with healthy dietary intakes.

Regarding agreeableness and conscientiousness our findings are similar to previous results, as high agreeableness and conscientiousness have both been associated with Mediterranean-style and health-aware diets [7]. Being conscientious has been associated with diets low in high-fat snacks and high in fruits and vegetables [32] and other favorable health behaviors such as exercising [2], [3]. A large meta-analysis showed high conscientiousness to be associated with beneficial health behaviors, such as less smoking, more physical activity and healthier dietary habits [2]. Previously, also the combination of low conscientiousness and high neuroticism has been associated with other unfavorable health behaviors, such as lack of exercise and smoking [33] and being predictive of premature mortality [12].

In this study, we also explored the combination of five traits, resilience, and found it to be associated with several healthy dietary intakes. To our knowledge, this is the first study to explore the associations of resilience and the whole diet. The two previous studies exploring the association of resilience and health behaviors used telephone interviews to assess diet and the measure of fruit and vegetable intakes was limited to the previous day [21], [22]. In these studies, resilience was associated with favorable health behaviors, such as exercising more, smoking less, and being more likely to consume ≥5 portions of fruits and vegetables a day and drinking less alcohol. The fact that we were able to observe significant differences between resilient and non-resilient subjects in their food and nutrient intakes gives us reason to assume that resilience is a notable aspect of personality affecting people’s dietary intake. Being a combination of five traits, resilience gives us more information than a single trait alone. The magnitude of the differences in dietary intake in resilient subjects vs. non-resilient subjects was pronounced as e.g. the difference in the combined vegetable and fruit intake was 110.3 g/d.

The strengths of our study include a large study population of men and women. The FFQ we used and the Finnish version of the NEO-PI have both been validated and used in several studies [23], [24], [26]. A limitation was that the subjects answered the NEO-PI around two years later than the FFQ. However, personality is stable in adulthood [34] enabling us to draw our conclusions. This study being cross-sectional does not allow drawing conclusions of causality.There is a possibility for measurement error as the subjects self-reported their dietary intakes. As usual, subjects tend to overestimate intakes of foods considered healthy and underreport intakes of foods considered unhealthy. This is especially true for women who tend to overestimate their fruit and vegetable intake [23] and for obese subjects, who often underreport intakes of energy dense foods. Including a broader age range of subjects would enable us to generalize the results to other age groups. We ran multiple statistical tests, but applied Bonferroni corrections in order to reduce Type I error. The results should be replicated in further studies.

We conclude that, as hypothesized, the personality traits, neuroticism, extraversion, openness, agreeableness and conscientiousness are associated with food and nutrient intake, and the effect is pronounced in resilient subjects as they showed several favorable dietary intakes. Resilience turned out to be a useful construct and resilience-diet associations should be studied further in different populations and age groups.
